# Prolamin Content and Grain Weight in RNAi Silenced Wheat Lines Under Different Conditions of Temperature and Nitrogen Availability

**DOI:** 10.3389/fpls.2020.00314

**Published:** 2020-03-20

**Authors:** Miriam Marín-Sanz, María J. Giménez, Francisco Barro, Roxana Savin

**Affiliations:** ^1^Department of Plant Breeding, Institute for Sustainable Agriculture (IAS-CSIC), Córdoba, Spain; ^2^Department of Crop and Forest Sciences, University of Lleida, Lleida, Spain

**Keywords:** gluten proteins, heat stress, transgenic lines, celiac disease, *Triticum aestivum*

## Abstract

Temperature and nitrogen (N) availability are two important environmental factors that may produce important changes in grain composition during grain filling of bread wheat. In this study, four wheat lines with the down-regulation of gliadins by means of RNA interference (RNAi) have been characterized to determine the effect of thermal stress and N availability on grain weight and quality; with focus on gliadin and glutenin protein fractions. Grain weight was reduced with heat stress (HS) in all RNAi lines, whereas gliadin content was increased in the wild-types. With respect to gliadin content, RNAi lines responded to HS and N availability differently from their respective wild-types, except for ω-gliadin content, indicating a very clear stability of silencing under different environmental conditions. In a context of increased temperature and HS events, and in environments with different N availability, the RNAi lines with down-regulated gliadins seem well suited for the production of wheat grain with low gliadin content.

## Introduction

Grain yield and quality are critical for wheat breeding and management. Both traits are determined during the grain-filling phase. Understanding the processes affecting grain weight and quality during grain filling is important for improving breeding and management strategies. Grain filling is commonly partitioned into three phases: the lag phase, the effective grain filling period, and the maturation drying phase (Egli, 1998). The lag phase is a period of active cell division, characterized by a rapid increase in water content with almost no dry matter accumulation. Grain dry weight then increases rapidly during the effective grain filling period until the maximum dry weight is attained, after which it remains approximately stable while the grain dries. During the effective grain filling period, starch and proteins are deposited in the endosperm (Jenner et al., 1991). It has been shown that the rate of their deposition is controlled by the source-sink balance (Fischer et al., 1977). Approximately 80% of total proteins in wheat grain are gluten (also termed prolamins) whereas the remaining 20% is composed of non-gluten proteins (NGPs) – mainly albumins and globulins (van den Broeck et al., 2009; Wen et al., 2012). Wheat gluten is able to form a network responsible for the viscoelastic properties of wheat flour since it allows the retention of carbon dioxide released during fermentation (Shewry, 2009). Gluten proteins can be further divided into two fractions: glutenins and gliadins (Lafiandra and Kasarda, 1985; Shewry, 2019). The glutenins form polymers linked by inter-chain disulfide bonds, they are insoluble in alcohol solutions, and can be divided according to their mobility in sodium dodecyl sulfate polyacrylamide gel electrophoresis (SDS-PAGE) into high molecular weight (HMW) and low molecular weight (LMW) glutenins. The gliadins are monomeric proteins, soluble in alcohol, and divided into three groups according to their mobility by electrophoresis in polyacrylamide gels at acidic pH (A-PAGE); ω, α and γ-gliadins. The glutenins are responsible for wheat dough elasticity and strength, while gliadins are important for viscosity and extensibility (Shewry and Halford, 2002; Shewry et al., 2003). Gluten proteins, particularly the gliadin fraction, are the primary factors responsible for triggering celiac disease (CD), since they contain the most immunogenic CD epitopes (Arentz-Hansen et al., 2000, 2002; Shan et al., 2002; Molberg et al., 2003; Ludvigsson et al., 2013; Box 1).

**BOX 1.** Celiac disease (CD) is a chronic enteropathy that results from the ingestion of gluten proteins present in wheat, and other similar proteins in barley and rye (Trier, 1998; Sollid, 2002). After ingestion of gluten, lesions form in the small intestine, characterized by flattening of the microvilli, hyperplasia of crypt cells, and infiltration of leukocytes (Sollid, 2002). As a result, symptoms such as diarrhea and malabsorption of food appear among others, since the spectrum of symptoms can be very broad. The immune response is triggered by the activation of CD4 T cells when they recognize the gluten peptides presented by serotypes HLA-DQ2 and HLA-DQ8. The presence of gluten peptides can be detected by the activity of the tissue transglutaminase 2 enzyme from the intestinal mucosa (Sollid, 2002; Sollid et al., 2012; Gayathri and Rashmi, 2014). CD is present throughout the world and the prevalence in the United States is around 1%, as in Europe, with the highest estimates in Finland and Sweden, and the lowest in Germany (Catassi et al., 2014). Gluten is present in many food products as the main element or as an additive. So far, the only possible treatment for CD is to follow a gluten-free diet for life (Sollid, 2002). The increase in the incidence of the disease was associated with the duration of exposure to gluten (Ventura et al., 1999), which increases the need to obtain wheat with a reduced content of proteins immunogenic for celiac sufferers.

In addition to CD, there are other pathologies related to wheat: (i) allergies as wheat-dependent exercise-induced anaphylaxis (WDEIA) – induced by the ω-5 gliadins and the HMW- (Morita et al., 2007; Morita et al., 2009), or baker’s asthma associated with non-specific lipid transfer proteins (Brant, 2007; Palacin et al., 2007); and (ii) non-celiac wheat sensitivity (NCWS) (Gibson et al., 2017), with an estimated prevalence ranging from 0.6 to 13% of global population (Aziz et al., 2016). Most of the allergens and proteins related to wheat pathologies have been mapped to the bread wheat Chinese Spring reference genome (RefSeq v1.0, International Wheat Genome Sequencing Consortium) (Appels et al., 2018) contributing to the knowledge of these diseases (Juhász et al., 2018). Moreover, there is a broad study on wheat allergens and CD peptides that allows their identification and composition for diagnostic assays by liquid chromatography-tandem mass spectrometry (Lexhaller et al., 2019).

RNA interference-based (RNAi) techniques are ideal for the down-regulation of specific protein fractions related to CD. Using this technology, γ-gliadins were silenced in two lines of bread wheat, providing reductions of up to 80% in this gliadin fraction (Gil-Humanes et al., 2008). Subsequently, the same workers used chimeric interference RNAs capable of silencing the genes from all the three groups of ω, γ and α-gliadins, to obtain several lines of two wheat genotypes with major reductions (in some cases up to 90%) in total gliadin content (Gil-Humanes et al., 2010). The crossing of the silenced lines with commercial varieties of wheat has allowed the obtention of new lines that effectively express the fragment of silencing in different genetic backgrounds, both to silence γ-gliadins (Gil-Humanes et al., 2012) and total gliadin fractions.

Climate model projections suggest that higher temperatures and heat stress events will become commonplace in most regions where grain crops are produced (Meehl and Tebaldi, 2004). Deleterious effects of high temperature on crop yield and quality are well documented in the literature (e.g. Barnabás et al., 2008). It is also well known that temperate species, such as wheat, maximize their vegetative growth during the period of colder temperatures, and the grain develops as temperature rises. Much works have studied the effects of very high temperature – moderate and short periods – during grain filling in wheat (Wardlaw et al., 2002; Farooq et al., 2011; Nuttall et al., 2018). Typically, high temperature during the grain filling linear phase results in the reduction of grain weight, mainly due to the decrease of soluble starch synthase activity under heat stress (Hawker and Jenner, 1993), reducing starch accumulation (e.g. Bhullar and Jenner, 1986). Simultaneously, heat stress (HS) not only increases protein percentage (Stone, 2001; Wardlaw et al., 2002), but also affects the synthesis of the different prolamin fractions and their ratios, which are responsible of the bread quality (Blumenthal et al., 1993; Stone, 2001).

Nitrogen (N) fertilization is one of the most common management practices used by farmers to improve yields. Consequently, there have been many studies analyzing yield (Foulkes et al., 1998; Barraclough et al., 2014) and protein content (Fischer et al., 1993) in response to soil N availability in wheat. In addition, several studies reported the effects of N on the types of proteins being synthesized during grain filling (Pechanek et al., 1997; Daniel and Triboi, 2000; Johansson et al., 2013), indicating that the synthesis of proteins in cereals is clearly influenced by temperature and N condition under which grain filling proceeds. However, genotypic variability can be found in all these responses (Saint Pierre et al., 2008; Elbashir et al., 2017); and even in the response to interactions between heat and N (Elía et al., 2018; Slafer and Savin, 2018). Therefore, determining the effects of heat and N availability on wheat genotypes with contrasting protein composition are of particular interest in the understanding grain protein distribution and its influence on grain weight and quality.

RNAi lines with low gluten content were subjected to various N and sulfur treatments to study the stability of the gliadin silencing under different fertilization conditions (García-Molina and Barro, 2017). In relation to N, this study showed that the RNAi lines had consistently lower gliadin levels than the wild-type across different N-fertilization regimes, but also that the level of gliadins in RNAi lines was sometimes significantly increased when N availability increased. In that study, N was applied when it would strongly affect grain number and yield and, therefore, may have diluted the availability of N-compounds during grain filling (i.e. more N available for absorption had to be shared between much higher grain numbers). As late N fertilization can be used to maintain green tissues during grain filling and to increase overall N content of the grains (Blandino et al., 2015), it may be relevant to explore whether the response of the RNAi lines would be even more marked. As mentioned before, grain filling is significantly affected by HS which would also favor the synthesis of proteins compared with that of starch (Barlow et al., 2015). It would thus be of interest to determine whether the synthesis of proteins in general, and gliadins in particular, in these RNAi lines is affected by the combinations of high temperatures and N availability during grain filling.

In this context, the objective of the present work was to determine the effects of contrasting temperature and N availability conditions on the silencing of gliadins. Thus, grain weight, total protein content and gluten protein distribution were studied in a set of RNAi lines and their respective wild-types. The aim was to determine to what degree the silencing of the synthesis of gliadins depends on environmental conditions, which is important for progressing in the development of low-gliadin wheat varieties suitable for new dietary approaches for gluten-related disorders.

## Materials and Methods

### Plant Material, Chamber Experiment and Treatments

Six lines of bread wheat were used: BW208, D770, D793, Gazul, J631, and M959. BW208 is a line from CIMMYT and Gazul is a commercial variety, and both were used as wild-types. D770 and D793 are lines derived from BW208 with RNA interference (RNAi) silencing of all gliadin fractions (Gil-Humanes et al., 2010). J631 and M959 are derived from crossing the Gazul genotype and line D770. Lines J631 and M959 were backcrossed at least four times with Gazul, always selecting the silencing character and the high and low molecular weight glutenins of Gazul, so that both silenced lines maintain the glutenin profile of this parent line.

We carried out a chamber experiment involving six wheat lines (two wild-type cultivars and four RNAi lines), two temperature treatments (control and heat stress, HS) during the linear phase of grain filling period, and two nitrogen (N) availabilities with three replicates, each replicate was composed of 6 plants (all in all there were 18 plants per genotype × N × HS; i.e. 216 plants per chamber). Two seeds were sown in pots (270 cm^3^) filled with a mixture of 30% peat and 70% soil. After emergence, one plant was left in each pot.

Plants were grown outdoors until heading when all pots were placed in a growth chamber set at 20/15°C. Different temperature treatments were imposed from 10 days after anthesis (DAA) during 10 consecutive days (Supplementary Figure 1). The control was set at 25/18°C in a chamber and the HS treatment to 40/18°C in another chamber. Minimum and maximum temperatures of 18 and 25 or 40°C were maintained for 8 and 6 h, respectively (Supplementary Figure 1). After the 10 days of treatment, temperatures were set to 25/18°C until maturity.

Chambers were set under long-day conditions (16 h). Pots inside the chambers were rearranged approximately once a week to minimize the effects of possible differences in microenvironment at different positions within each chamber. Pots were watered regularly to avoid water stress. N (9 mg per pot) was applied as urea diluted in all pots at flag leaf appearance (DC 4.5, Zadoks et al., 1974). At heading (DC 5.9), half of the plants received another dose of N (21 mg per pot).

### Grain Weight and Total Protein Determination

At maturity, 18 plants per treatment were sampled. Mature grain weight was determined as the average of all grains from the main spikes harvested. Total grain protein content was determined by Dumas methodology (Dumas, 1831).

### Prolamins Quantification by RP-HPLC

For gliadin and glutenin extraction, two grains from three different plants of each line and treatment were weighed and ground using a ball mill, and sequentially extracted following a previous protocol (Pistón et al., 2011) adapted to small samples. Briefly, gliadins were extracted stepwise three times with up to 400 μL of 60% (v/v) ethanol. Samples were centrifuged, and the supernatants collected, mixed together and filtered. The insoluble pellet was re-suspended in 50% (v/v) 1-propanol, 2 M urea, 0.05 M Tris–HCl (pH 7.5), and 2% (w/v) DTT for glutenin extraction, incubated for 30 min at 60°C and centrifuged stepwise three times. For each sample, the three collected supernatants were mixed together and filtered. The protein extracts were used for gliadin and glutenin quantification by Reverse-Phase High-Performance Liquid Chromatography (RP-HPLC, 1200 Series Quaternary LC System liquid chromatography from Agilent Technologies) with a DAD UV-V detector at 210 nm. A 25 cm long column LiChrospher^®^ 100 RP8 (5 μm) (Merck) was used at 50°C and a sample volume equivalent to 2 mg of flour was injected. The flow rate was 0.5 mL⋅min^–1^. Mobile phase consisted in a mixture of Acetonitrile (ACN 0.1% TFA) and 0.1% aqueous TFA in a linear gradient (0 min 26% ACN, 60 min 54% ACN). The absolute amount of protein was calculated using bovine serum albumin protein as standard (BSA; BSA ≥ 98%, fraction V. Sigma-Aldrich, St Louis, MO, United States cat. no. A3294) (Supplementary Figure 2). The intervals of retention time used for the separation of prolamin fractions peaks are indicated in Supplementary Figure 3 according to Wieser et al. (1998). The integration of the peaks was performed automatically by RP-HPLC software with minor modifications if necessary.

### Non-gluten Proteins (NGPs) Determination

The NGPs content was calculated by the difference between the total protein and prolamin content (glutenins and gliadins) for each line. The total protein content (μg/mg) was calculated from the percentage of N obtained by Dumas using the wheat conversion factor (5.83) (Merrill and Watt, 1973).

### Data Processing

The retention time (min) and area (mAU) output of the RP-HPLC software was imported into a house developed software made in Python v2.7^1^ to obtain the average values from the transformed technical repeats using the following formulas, that processes the hundreds of output files in a single run. The output of the software is a file with Microsoft Excel format.

$$\text{Protein}\,\left(\mu \,g\,{\left(\mathrm{mg}\,\text{of}\,\mathrm{flour}\right)}^{-1}\right)=0.0005\cdot \text{Area}\,\left(\mathrm{mAU}\right)\,\frac{{\text{V}}_{\text{extraction}}\,\left(\mu \,L\right)}{{\text{V}}_{\text{injection}}\,\left(\mu \,L\right).\text{Grain}\,\mathrm{weight}\,\left(\mathrm{mg}\right)}$$

The integration of the profiles, to obtain the area of each peak, and the subsequent transformation using the formula described, allow estimating the amount of protein for the samples. The arithmetic mean of the three technical repetitions was used for the variance analysis.

### Statistical Analysis

To determine the effect of HS and N multifactorial univariates ANOVA were performed. Two variants of this model were tested: in the first, genotype, temperature, nitrogen and their interactions were independent variables, while grain weight and protein fractions were dependent variables. It was used to determine the general effect of the treatments on all genotypes. The second, has the same factors and variables, but it was performed for wild-types and RNAi lines separately to determine the effect of the treatments on each of these groups. Principal Components Analysis, PCA, was carried out with grain weight, total gliadin and its fractions, total glutenin and its fractions, and total prolamin as variables to evaluate their contribution to the model variance. The software used for the statistical analysis was R v 3.5.1 (R Core Team, 2018).

## Results

### Heat Stress and Nitrogen Treatment Effects on Grain Weight and Total Protein

**FIGURE 1:**
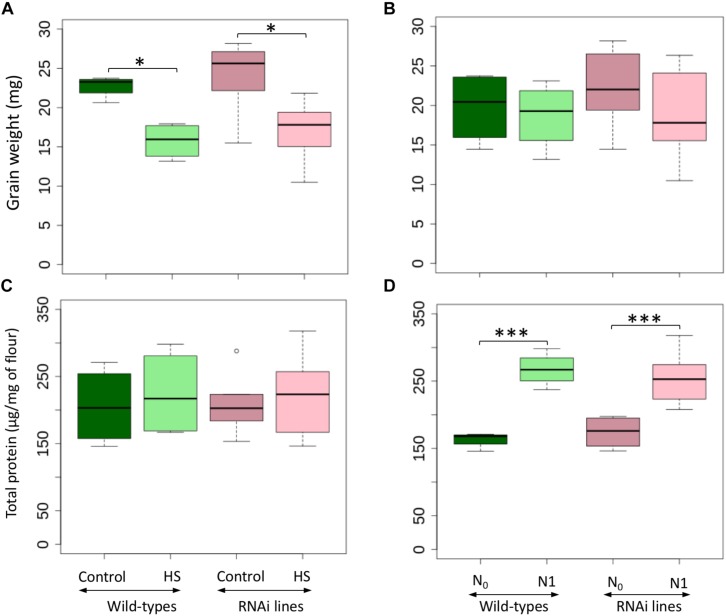
Grain weight and total protein content for wild-types and RNAi lines under control and heat stress temperature treatments **(A,C)** and nitrogen availability **(B,D)**. N_0_: no N application after heading, N_1_: N application after heading; control: 25/18°C during whole grain filling period, Heat stress (HS): 40/18°C for 10 days during grain filling period. The black line represents the median value. ^∗^ above the bars indicates significant difference (^∗^*P* ≤ 0.05; ^∗∗∗^*P* ≤ 0.001) between treatments according to the variance analysis.

Grain weight was significantly decreased in both wild-types and RNAi lines by heat stress (HS) (Figure 1A, Table 1, and Supplementary Table S1). Additional applications of nitrogen (N) had no significant effect on the RNA interference (RNAi) lines or wild-types (Figure 1B and Table 1). No significant differences were found in total grain protein content among all genotypes (Table 1). HS for a short period did not significantly modify the total protein content for both wild-types and RNAi lines (Figure 1C), but the late application of N (N_1_) resulted in a significant increase of the total protein for both the wild-types and the RNAi lines (Figure 1D).

**TABLE 1:** Significance of the variance of effects of genotype (6 lines: BW208, Gazul, D770, D793, J631 and M959), temperature (2 levels: Control temperature and heat stress), nitrogen (2 levels: N_o_ and N_1_) and their interactions for each variable studied (grain weight, protein fractions and protein ratios).

Variables	Factors	*P*-value	Variables	Factors	*P*-value
Grain weight	G	0.002082**	HMW	G	0.16009
	T	3.25E-05***		T	0.09833
	N	0.003541**		N	0.05425
	GxT	0.604707		GxT	0.55593
	GxN	0.092625		GxN	0.84124
	TxN	0.678413		TxN	0.1672

Total protein	G	0.6512291	LMW	G	0.005985**
	T	0.2856815		T	0.142497
	N	0.0008094***		N	0.104511
	GxT	0.9285325		GxT	0.051446
	GxN	0.5782825		GxN	0.333028
	TxN	0.2003991		TxN	0.929684

ω-gliadins	G	6.27E-05***	Total glutenins	G	0.39305
	T	0.07043		T	0.08163
	N	3.81E-05***		N	0.04616*
	GxT	0.00102**		GxT	0.34164
	GxN	0.06651		GxN	0.65589
	TxN	0.059		TxN	0.24875

α-gliadins	G	1.30E-05***	Ratio GLI/GLU	G	0.0006601***
	T	0.075602		T	0.0555856
	N	0.001394**		N	0.3908054
	GxT	0.014791*		GxT	0.1579138
	GxN	0.010881*		GxN	0.2167633
	TxN	0.583088		TxN	0.9445682

γ-gliadins	G	3.01E-07***	Ratio GLI/TP	G	2.79E-05***
	T	0.1145912		T	0.87811
	N	0.1861917		N	0.009226**
	GxT	0.0005413***		GxT	0.024892*
	GxN	0.180311		GxN	0.072216
	TxN	0.9548613		TxN	0.242135

Total gliadins	G	1.88E-06***	Ratio GLU/TP	G	0.23479
	T	0.1784208		T	0.06773
	N	0.0004081***		N	0.08808
	GxT	0.0016167**		GxT	0.30135
	GxN	0.0127328*		GxN	0.45733
	TxN	0.8348028		TxN	0.17623

The *P*-value is presented for significant factors of each variable. *P ≤ 0.05, **P ≤ 0.01, ***P ≤ 0.001. G, genotype; T, temperature; N, nitrogen; HMW, high molecular weight; LMW, low molecular weight; ratio GLI/GLU, ratio total gliadin content/total glutenin content; ratio GLI/TP, ratio total gliadin content/total protein content; ratio GLU/TP, ratio total glutenin content/total protein content; total proteins, total protein content in percent of nitrogen by Dumas. The degree of freedom (d. f.) of the variance analysis (N -1, N: number of observations) for the factors are: G, 5; T, 1; N, 1; GxT, 5; GxN, 5; TxN, 1. *P* < 0.05 are in bold.

### Heat Stress and Nitrogen Treatment Effects on Gliadins and Glutenins

**FIGURE 2:**
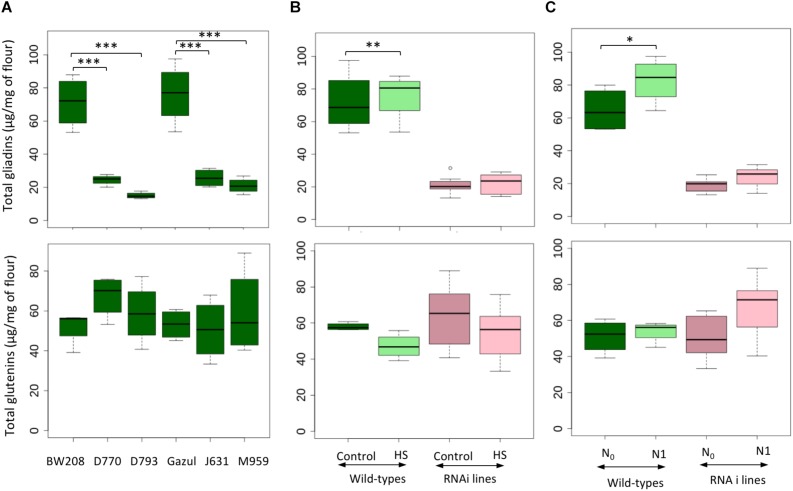
Content of total gliadin and total glutenin content for different genotypes with comparisons between RNAi lines and their wild-type by Dunnett’s test **(A)**, wild-types and RNAi lines under different temperature treatments **(B)** and nitrogen availability **(C)**. N_0_: no N application after heading, N_1_: application after heading; control: 25/18°C during whole grain filling period, Heat stress HS: 40/18°C for 10 days during grain filling period. The black line represents the median value. ^∗^ above the bars indicates significant difference (^∗^*P* ≤ 0.05; ^∗∗^*P* ≤ 0.01) between treatments according to the variance analysis.

We confirmed that total gliadin content was significantly higher in the wild-types than in the RNAi lines (Figure 2A and Table 1). Among RNAi lines, D793 had lower content of gliadins than that of the rest of RNAi lines (Supplementary Table S1). Both HS and the late application of N (N_1_) resulted in a significant increase in the total gliadin content for the wild-type lines, whereas no significant variation was observed for the RNAi lines (Figures 2B,C).

The degree of silencing of ω-gliadins was lower than that of the rest of the gliadin fractions (Supplementary Figure 4A and Supplementary Table S1). A significant increase in the content of ω-gliadins due to supplementary N was found on both wild-types and RNAi lines, while HS only had an effect on the wild-types (Supplementary Figures 4B,C and Table 1). An overall effect of N level on the α-gliadin content (Supplementary Figure 4C and Table 1), as well as on the total gliadin content (Figure 2C), was observed in the wild-types, but the RNAi lines did not show this effect. HS treatment had no effect on the α-gliadin content for wild-types and RNAi lines (Supplementary Figure 4B). Conversely, γ-gliadin content was not affected by N availability, but a decrease in the amount of this fraction under HS was seen in wild-types, but not in the RNAi lines (Supplementary Figure 4B and Supplementary Table S1). Overall, the RNAi line D793 showed the highest reduction in α- and γ-gliadins (Supplementary Figure 4A and Supplementary Table S1).

The content of glutenins at grain maturity (Supplementary Table S1) was not significant higher for most RNAi genotypes than that of the wild-type lines (Figure 2A). The content of HMW was not statistically different between wild-types and RNAi lines (Supplementary Figure 5A and Table 1). HS had no effect on the HMW fraction, and for N treatment, RNAi lines tend to have a higher amount of HMW. In contrast, the LMW fraction was decreased in most of the RNAi lines in comparison to that of the wild-types (Supplementary Figure 5A, Table 1, and Supplementary Table S1). In addition, LMW content was affected by HS and N availability; in the wild-types LMW proteins decreased under HS; in RNAi lines LMW fractions increased at higher N availability (Supplementary Figures 5B,C and Supplementary Table S1).

**FIGURE 3:**
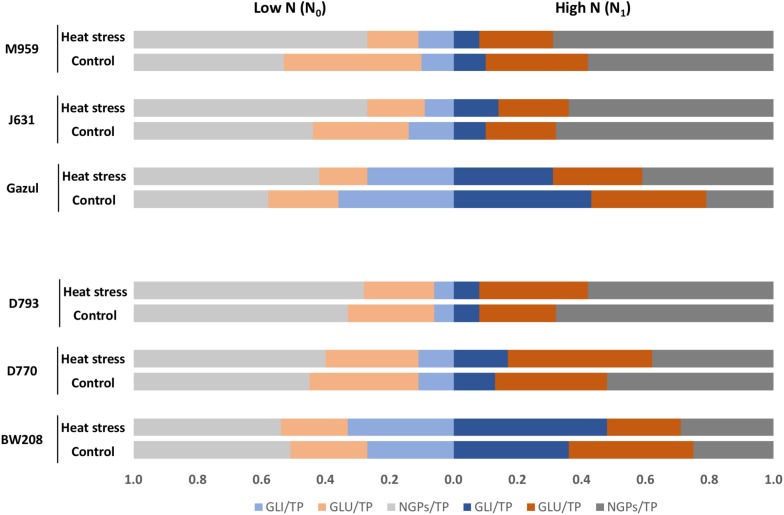
Total gliadin/total grain protein ratio (GLI/TP), total glutenin/total grain protein ratio (GLU/TP) and NGPs/total grain protein ratio (NGPs/TP) between treatments and genotypes. The ratios were obtained with mean values for protein content. Low N: no N application after heading, High N: N application after heading; control: 25/18°C during whole grain filling period, Heat stress HS: 40/18°C for 10 days during grain filling period.

The total gliadin/total protein ratio (GLI/TP) confirms that RNAi lines have lower gliadin content than the wild-types, particularly line D793 whatever experimental conditions. However, a lower total glutenin/total protein ratio (GLU/TP), was seen in wild-types than in RNAi lines without additional N supply, and in BW208 under HS with N_1_ than in RNAi lines (Figure 3 and Table 1).

HS and N availability modified the GLI/TP ratio in the wild-type lines in different ways: it increased with N_1_ and HS in BW208 and decreased in Gazul (Figure 3). In contrast, minor effects were found in the GLI/TP ratio for the RNAi lines (Figure 3). Regarding the GLU/TP ratio, it was also modified by HS and N availability; it was strongly decreased in Gazul wild-type and RNAi derived lines under HS treatment and N_0_, while only a minor effect on lines with BW208 background was observed under those conditions; GLI/TP ratio was increased in BW208 RNAi lines under HS and N_1_, with only but minor effects in Gazul RNAi lines (Figure 3).

**FIGURE 4:**
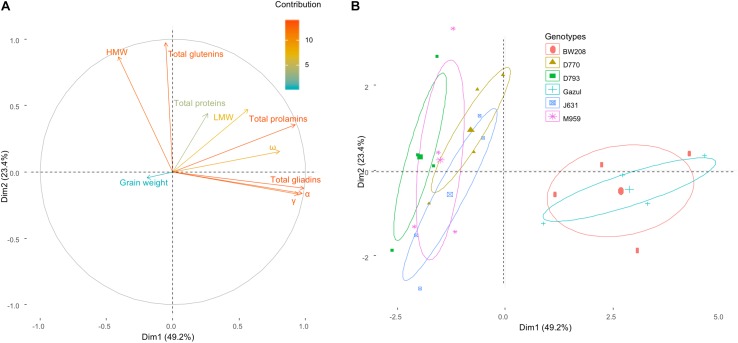
Principal Components Analysis (PCA). Effect of genotypes, temperature and N availability treatments on the variation of the protein fractions and grain weight **(A)**. The high values in the color scale indicates a high contribution to the PCA. The direction and the size of the vectors indicate the relationship between all variables and their contribution to each axis. **(B)** Individuals are represented on the PCA axes with the 95% confidence ellipses showed for each genotype. The largest point for each genotype indicates the intersection of ellipse axes.

A Principal Component Analysis (PCA) was carried out, considering the effect of genotypes, temperature and N availability treatments on the variation of the protein fractions and grain weight (Figure 4A). Among the gliadin fractions, ω-gliadin and α-gliadin fractions contribute less and more, respectively, to the variance of the model. HMW proteins were the glutenin fraction that contributed most (Figure 4A). Gliadin and glutenin contents varied in perpendicular directions, indicating an independent behavior of both families of proteins (Figure 4A). The wild-types are separated from the RNAi lines (Figure 4B). The ellipses of 95% confidence level of each genotype indicated that there was a strong association between the variation of the glutenins and the silenced genotypes, and on the other hand, between the variation in the prolamins and the wild-types. D770 and J631 tend toward the direction of variation of the LMW proteins, and D793 and M959 toward that of the total glutenins (Figure 4). It is interesting to note that the variation in grain weight was independent of variations in the different protein fractions.

## Discussion

Grain weight, total grain protein, and prolamin content under control temperature and N_0_ (control conditions) resulted in values similar to those previously reported for gliadin down-regulated lines (Gil-Humanes et al., 2010; Pistón et al., 2013). The decrease of the gliadin/total protein (GLI/TP) ratio in the RNA interference (RNAi) lines could be explained by the increase of the NGPs, as the glutenin/total protein (GLU/TP) ratio was higher in RNAi lines. In previous studies with these and other RNAi lines, protein compensation was observed (Altenbach et al., 2014; García-Molina and Barro, 2017) with increments in non-gluten proteins (NGPs) such as serpins, triticins and globulins (Gil-Humanes et al., 2011; Barro et al., 2016).

Brief heat stress (HS) events during the grain filling period generally result in a decrease in grain weight (Bhullar and Jenner, 1986; Savin et al., 1999). The range of variation depends on the genotype, timing and severity of HS (Balla et al., 2019). In the present study, we found a reduction in grain weight of 30% on average under HS and different availability of nitrogen (N). Grain weight was reduced by 35% for lines with BW208 genetic background, and about 24% for Gazul and its RNAi derived lines. This reduction could be mainly due to an extreme dependence on the temperature of starch synthesis, and an irreversible effect of HS on starch production after only a few days or even a few hours per day under control (Daniel and Triboi, 2000; Triboï et al., 2003; Spiertz et al., 2006; DuPont et al., 2006b; Liu et al., 2011; Hurkman et al., 2013) or field conditions (Savin et al., 1996; García et al., 2016; Elía et al., 2018). The percentage of grain protein generally increases under moderately high and very high temperatures (Stone, 2001; Wardlaw et al., 2002), either by a reduction of starch greater than the accumulation of protein, or by a reduction in starch without no change in protein accumulation. However, this response may not always occur for different genetic and environmental backgrounds (Graybosch et al., 1995). Interestingly, in the present study, HS resulted in a higher proportion of gliadin fraction in the wild-types. Other studies reported no effect of HS on total prolamins or even found a decrease with HS (DuPont et al., 2006b; Hurkman et al., 2013). However, Daniel and Triboi (2000) studied each fraction of gliadins and found that the proportion of ω- and α-gliadins increased with HS while γ-gliadins decreased, as found in the wild-types in the present study with the exception of α-gliadins. Also, in this work, grain protein content was increased under HS when post-anthesis N availability was higher (N_1_), whereas the total prolamin content of the wild-types differed in the response to HS. The RNAi lines, regardless of their genetic background, did not respond to temperature treatments for the total gliadin content, and for the gliadin fractions. This is an indication that these lines have robust gliadin silencing, independently of the temperature environment. Several authors have described a slight increase in LMW and HMW glutenin fractions with HS (DuPont et al., 2006a, b). Evidence has been also reported that the effect of HS is to cause a reduction in the size of glutenin polymers (e.g. Naeem et al., 2012). Nevertheless, in the present work, total glutenin and their fractions content were not modified significantly with HS in any of the genotypes, except for LMW in wild-types, but there is a non-significant decrease in all fractions in wild-types and RNAi lines.

Under higher N availability (N_1_), both wild-type and RNAi lines increase total grain protein, as previously described for other wheat genotypes (Daniel and Triboi, 2000; Triboï et al., 2003). Moreover, when increasing and splitting N doses, grain protein concentration increases and protein composition changes by increasing glutenin fractions (Xue et al., 2016). The response to N_1_ in wild-type lines in greater and in RNAi lines in lesser proportion, also confirmed that gliadin to glutenin ratio increase with N_1_ (Triboï et al., 2003). The content of ω-gliadins under N_1_ was increased, in comparison to N_0_, in the wild-types and RNAi lines, whereas the α-gliadin fraction was increased only in the wild-types as previously reported in D793 and other RNAi lines (Gil-Humanes et al., 2010; García-Molina and Barro, 2017). Total gliadin content did not increase in RNAi lines when additional N was supplied (N_1_), and this increase of ω-gliadins under N_1_ in RNAi lines has to be considered in further designing silencing constructs to improve their effectiveness since the ω-5 gliadins are related to wheat-dependent exercise-induced anaphylaxis (WDEIA) (Inomata, 2009; Morita et al., 2009) and ω-1,2 gliadins to CD (Tye-Din et al., 2010). However, the α-gliadins are reported as the major immunogenic complex in wheat, they contain three major celiac disease (CD) immunogenic peptides (Ozuna et al., 2015), and active peptides from this gliadin fraction were responsible for most of the immune response in patients with CD after eating wheat (Tye-Din et al., 2010). The α-gliadins were strongly reduced in the RNAi lines, and this was not affected either by HS or N application. Although some authors have indicated that LMW proteins decrease with high N availability at moderate temperatures (DuPont et al., 2006b; Hurkman et al., 2013), we found that LMW content increased in RNAi lines when additional N was supplied (N_1_). In contrast, the response in the HMW fraction and total glutenin content under N_1_, was not statistically significant in any of the genotypes.

## Conclusion

Wheat grain proteins are important for the breadmaking quality of wheat, but they are also related to human pathologies as celiac disease (CD) and other gluten intolerances. RNA interference (RNAi) technology has provided wheat lines with all the gliadin fractions strongly down-regulated. Heat stress (HS) and nitrogen (N) availability could affect the synthesis and deposition of proteins during grain filling. Wild-types and RNAi lines studied in this work responded similarly for total grain protein and the content of ω-gliadins to additional N supply, as well as for the grain weight under HS. While the wild-types increase their total gliadin content under HS or high N availability the RNAi lines did not. Interestingly, the α-gliadin content, the most CD immunogenic fraction, is unaffected in the RNAi lines under additional N supply, but it was increased in wild-types. Therefore, under the specific scenario of brief events of temperature increase or additional application of N, studied in this work, the RNAi lines demonstrated a high stability of down-regulation of gliadins. However, further evaluations under field conditions will be necessary to confirm that the silencing of gliadin fractions in RNAi lines can be maintained under different abiotic stress environments.

## Data Availability Statement

All datasets generated for this study are included in the article/Supplementary Material.

## Author Contributions

FB and RS designed the work. MM-S, MG, and RS carried out the work. All authors wrote the manuscript and approved the manuscript.

## Conflict of Interest

The authors declare that the research was conducted in the absence of any commercial or financial relationships that could be construed as a potential conflict of interest.

## Footnotes

1 https://github.com/MiriamMarinS/prolaminsQuantification
