# Supplementary material for: Prolamin Content and Grain Weight in RNAi Silenced Wheat Lines Under Different Conditions of Temperature and Nitrogen Availability
Source: Front Plant Sci. 2020 Mar 20;11:314. doi: 10.3389/fpls.2020.00314 (PMC7100604; doi:10.3389/fpls.2020.00314)
Supplement: Supplementary file 1 [file Data_Sheet_1.docx]

Supplementary Material

## Supplementary Figures

**Supplementary Figure 1.** Temperatures during the day and period of treatment imposition for the unheated control (blue, bottom lines) and the heated treatments (red, upper lines). Temperatures were determined hourly in control and heated experimental chambers for all wheat lines.


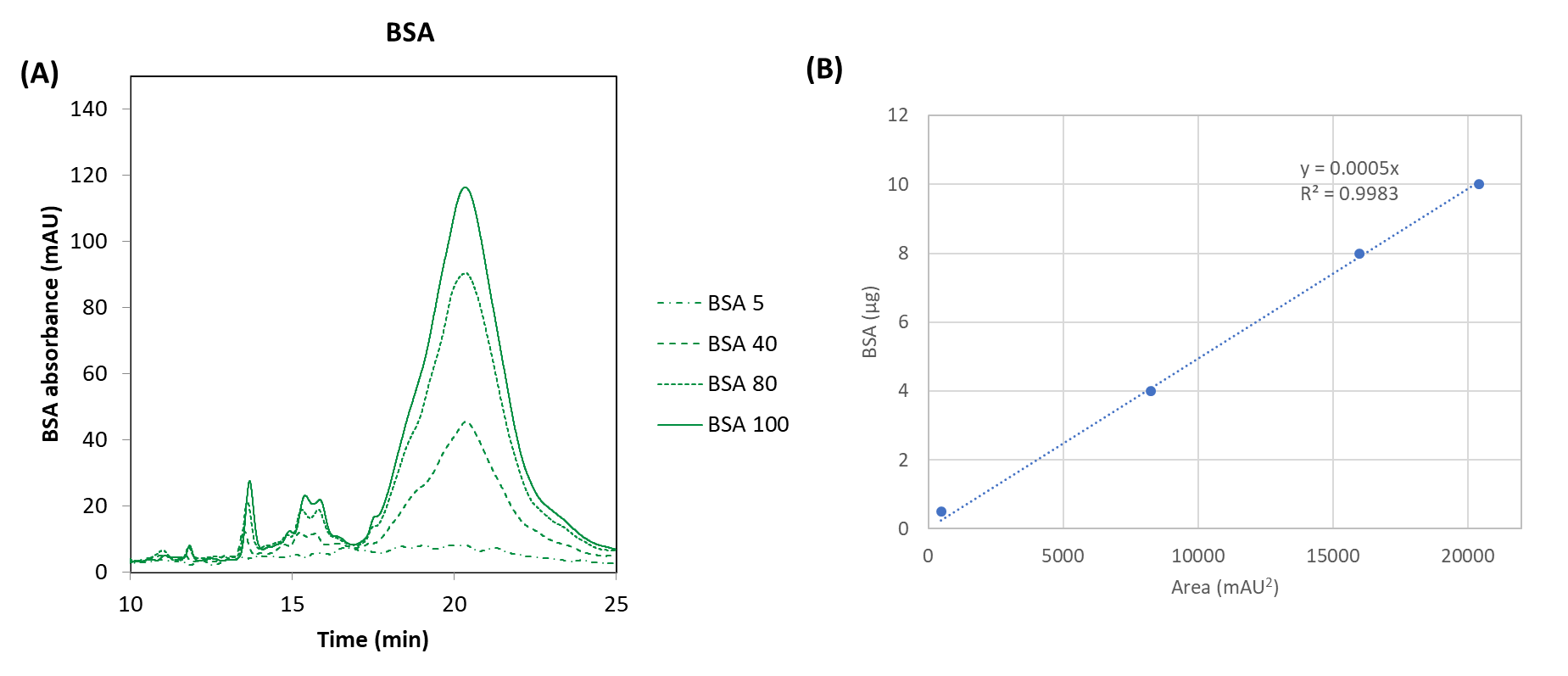


**Supplementary Figure 2.** RP-HPLC chromatograms of BSA used as standards. Three different volumes of solution of 0.1 µg/µL BSA protein dilluted in water were quantified (5 µL, 40 µL, 80 µL, 100 µL) (A). The linear regression between the BSA content and the area of the peak at 20 min has a R^2^ coefficient closer to 1 and the slope of the linear equation is the coefficient used for the data transformation formula (B).


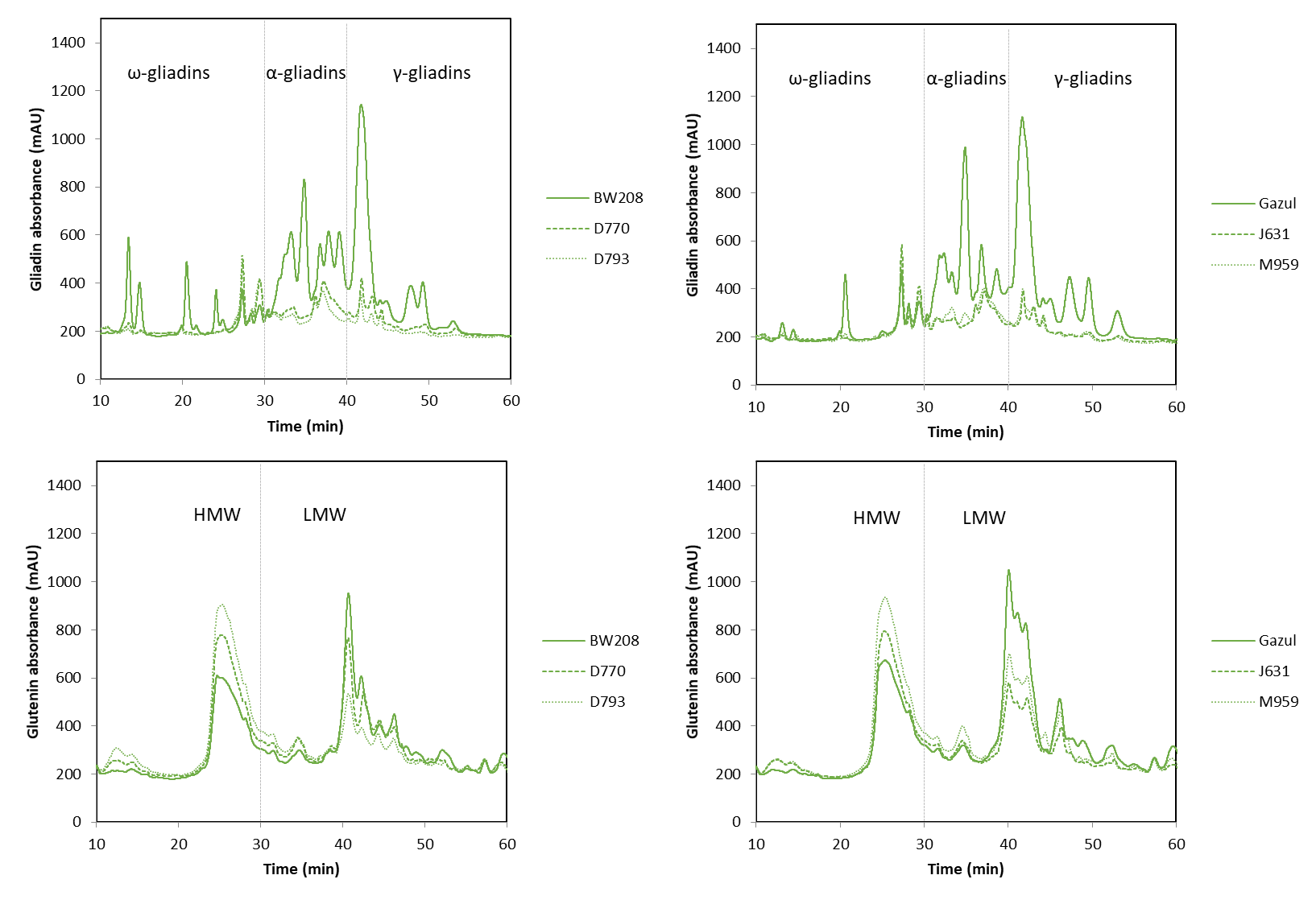


**Supplementary Figure 3.** RP-HPLC chromatograms of gliadin and glutenin fractions of all genotypes and control treatment conditions. N_0_: no N application after heading; control: 25/18 ºC during whole grain filling period. The signals were obtained by RP-HPLC software and represented with Microsoft Excel. The intervals of retention time used for the separation of prolamin fractions peaks are indicated according to Wieser et al. (1998).


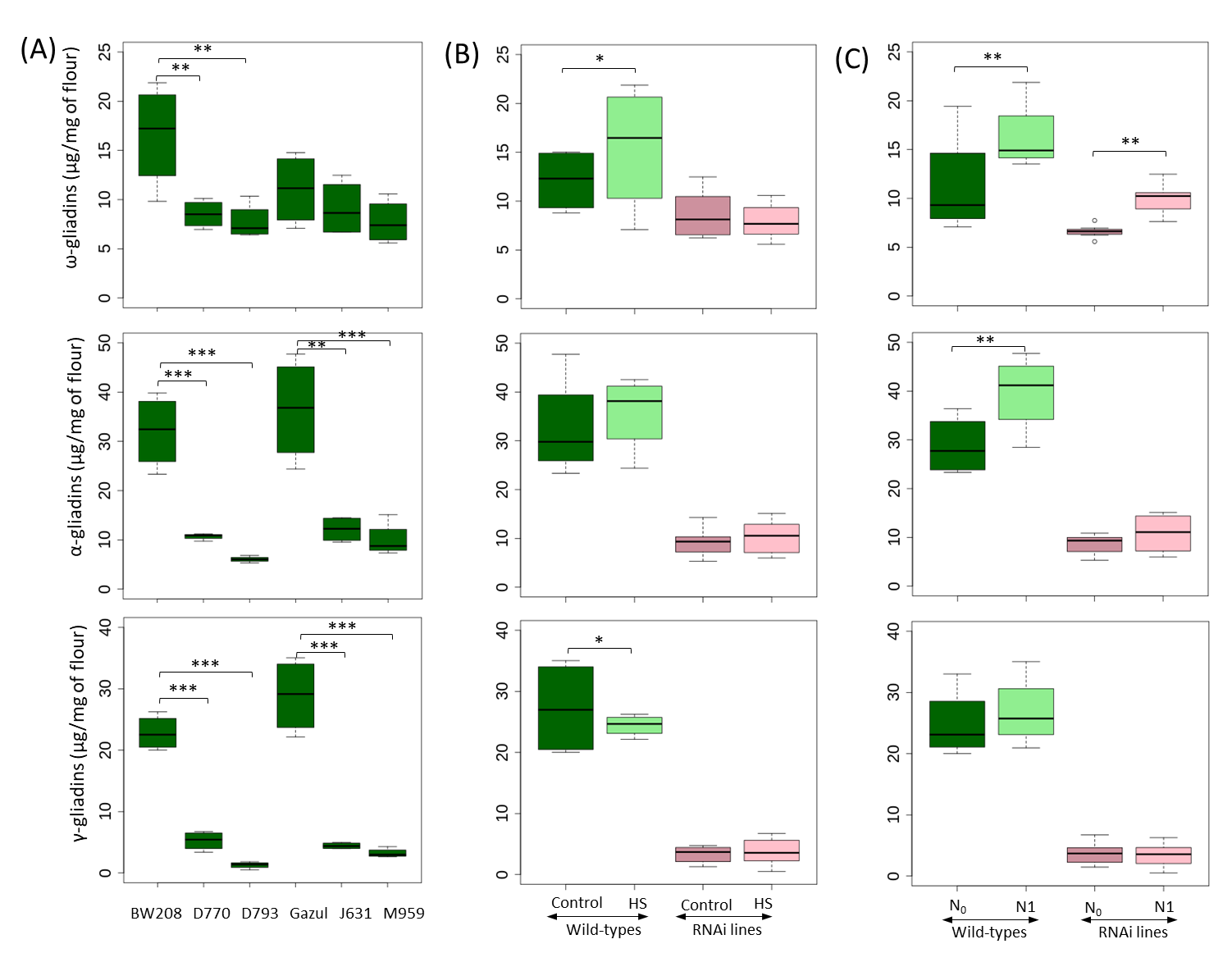


**Supplementary Figure 4.** Content of ω-, α- and γ-gliadins under Heat stress (HS) and N availability. (A) Comparisons between RNAi lines and their wild-type by Dunnett’s test, and for wild-types and RNAi lines under HS (B) and nitrogen availability (C). N_0_: no N application after heading, N_1_: N application after heading; control: 25/18ºC during whole grain filling period, HS: 40/18 ºC for ten days during grain filling period. The black line represents the median value. * above the bars indicates significant difference (*, *P* ≤ 0.05; **, *P* ≤ 0.01; ***, *P* ≤ 0.001) between treatments according to the variance analysis.

**
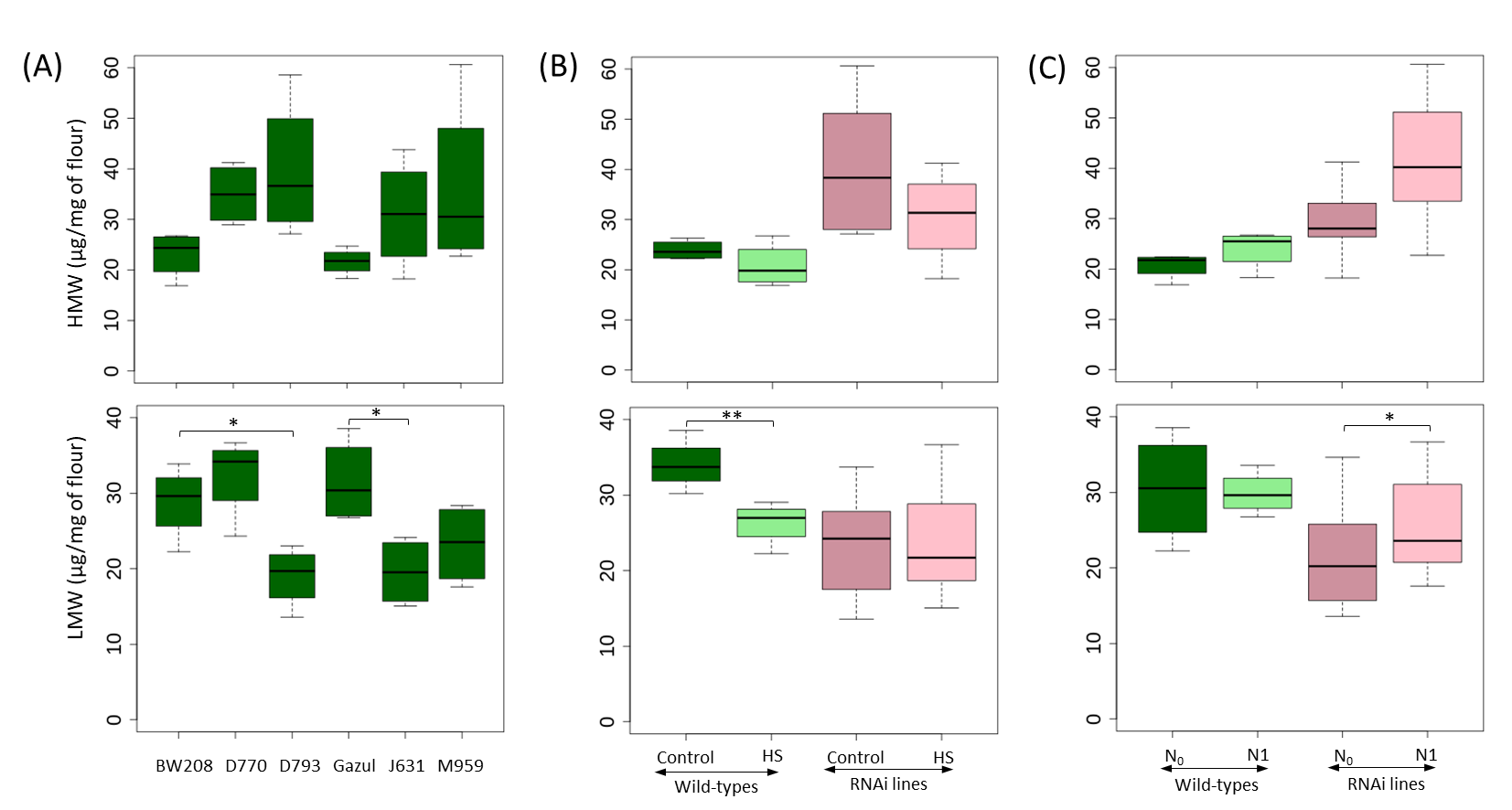
**

**Supplementary Figure 5.** Content of HMW and LMW under Heat stress (HS) and N availability. (A) Comparisons between RNAi lines and their wild-type by Dunnett’s test, and for wild-types and RNAi lines under HS (B) and nitrogen availability (C). N_0_: no N application after heading, N_1_: N application after heading; control: 25/18 ºC during whole grain filling period, HS: 40/18 ºC for ten days during grain filling period. The black line represents the median value. * above the bars indicates significant difference (*, *P* ≤ 0.05; **, *P* ≤ 0.01) between treatments according to the variance analysis.

## Supplementary Table

**Supplementary Table S1.** Mean values for total grain protein (µg/mg of flour), total gliadins and glutenins (µg/mg of flour), and their fractions (µg/mg of flour), and grain weight (mg) for each genotype under low (N_0_) or high nitrogen (N_1_), and under control temperature and Heat Stress.

| **Genotype** | **Temperature** | **Nitrogen** | **ω-gliadin** | **α-gliadin** | **γ-gliadin** | **Total gliadins** | **HMW** | **LMW** | **Total glutenins** | **Total prolamins** | **Total grain protein** | **Grain weight** |
| --- | --- | --- | --- | --- | --- | --- | --- | --- | --- | --- | --- | --- |
| BW208 | Control | N_o_ | 9.8 | 23.3 | 20.0 | 53.2 | 22.4 | 33.9 | 56.3 | 109.5 | 146.0 | 23.7 |
| D770 | Control | N_o_ | 7.0 | 9.8 | 3.4 | 20.1 | 28.9 | 24.3 | 53.3 | 73.3 | 153.3 | 27.9 |
| D793 | Control | N_o_ | 6.4 | 5.3 | 1.5 | 13.2 | 27.2 | 13.6 | 40.7 | 54.0 | 172.0 | 22.2 |
| Gazul | Control | N_o_ | 8.8 | 31.1 | 33.0 | 72.9 | 22.2 | 38.5 | 60.8 | 133.7 | 169.2 | 23.4 |
| J631 | Control | N_o_ | 6.7 | 9.6 | 4.0 | 20.3 | 27.2 | 16.3 | 43.5 | 63.8 | 195.6 | 28.2 |
| M959 | Control | N_o_ | 6.3 | 9.1 | 4.3 | 19.7 | 35.4 | 27.3 | 62.7 | 82.4 | 197.5 | 25.2 |
| BW208 | Control | N_1_ | 15.0 | 28.5 | 21.0 | 64.5 | 26.3 | 30.2 | 56.5 | 120.9 | 237.4 | 20.6 |
| D770 | Control | N_1_ | 9.3 | 10.9 | 4.6 | 24.8 | 41.3 | 33.7 | 75.0 | 99.8 | 223.4 | 22.1 |
| D793 | Control | N_1_ | 10.4 | 6.1 | 1.3 | 17.7 | 58.6 | 18.7 | 77.3 | 95.0 | 288.0 | 15.5 |
| Gazul | Control | N_1_ | 14.8 | 47.7 | 35.0 | 97.5 | 24.7 | 33.6 | 58.3 | 155.8 | 270.9 | 23.1 |
| J631 | Control | N_1_ | 12.5 | 14.3 | 4.8 | 31.5 | 43.8 | 24.2 | 67.9 | 99.4 | 223.4 | 26.1 |
| M959 | Control | N_1_ | 10.6 | 8.4 | 2.8 | 21.8 | 60.6 | 28.4 | 89.0 | 110.8 | 208.1 | 26.4 |
| BW208 | Heat stress | N_o_ | 19.4 | 36.4 | 24.1 | 79.9 | 16.9 | 22.2 | 39.1 | 119.1 | 167.0 | 14.5 |
| D770 | Heat stress | N_o_ | 7.7 | 10.9 | 6.7 | 25.3 | 30.7 | 34.6 | 65.4 | 90.7 | 146.4 | 19.0 |
| D793 | Heat stress | N_o_ | 6.6 | 6.8 | 1.8 | 15.2 | 41.3 | 20.7 | 61.9 | 77.2 | 180.2 | 14.5 |
| Gazul | Heat stress | N_o_ | 7.1 | 24.4 | 22.1 | 53.6 | 21.3 | 27.2 | 48.5 | 102.1 | 171.1 | 17.5 |
| J631 | Heat stress | N_o_ | 6.7 | 10.2 | 5.0 | 21.9 | 18.2 | 15.1 | 33.3 | 55.2 | 153.8 | 21.8 |
| M959 | Heat stress | N_o_ | 5.6 | 7.4 | 2.7 | 15.6 | 25.7 | 19.8 | 45.4 | 61.0 | 194.7 | 19.9 |
| BW208 | Heat stress | N_1_ | 21.9 | 39.9 | 26.2 | 88.0 | 26.8 | 29.1 | 55.8 | 143.8 | 263.2 | 13.2 |
| D770 | Heat stress | N_1_ | 10.1 | 11.3 | 6.3 | 27.7 | 39.2 | 36.7 | 75.8 | 103.5 | 261.0 | 15.6 |
| D793 | Heat stress | N_1_ | 7.6 | 6.0 | 0.5 | 14.1 | 32.0 | 23.0 | 55.0 | 69.1 | 252.2 | 10.5 |
| Gazul | Heat stress | N_1_ | 13.5 | 42.5 | 25.3 | 81.3 | 18.3 | 26.8 | 45.1 | 126.4 | 298.2 | 17.9 |
| J631 | Heat stress | N_1_ | 10.6 | 14.5 | 4.0 | 29.1 | 35.0 | 22.8 | 57.7 | 86.8 | 318.0 | 16.8 |
| M959 | Heat stress | N_1_ | 8.6 | 15.1 | 3.2 | 26.9 | 22.7 | 17.6 | 40.3 | 67.2 | 253.5 | 18.9 |
